# A Highly Efficient *Agrobacterium rhizogenes*-Mediated Hairy Root Transformation Method of *Idesia polycarpa* and the Generation of Transgenic Plants

**DOI:** 10.3390/plants13131791

**Published:** 2024-06-28

**Authors:** Hui Wang, Kaimao Cheng, Tongjie Li, Xiaoyu Lan, Li Shen, Huayan Zhao, Shiyou Lü

**Affiliations:** 1 State Key Laboratory of Biocatalysis and Enzyme Engineering, School of Life Sciences, Hubei University, Wuhan 430062, China; huiwang@stu.hubu.edu.cn (H.W.); 17764217061@163.com (K.C.); tongjieli@stu.hubu.edu.cn (T.L.); 2 Hubei Hongshan Laboratory, Wuhan 430070, China; 3 Shaanxi Agricultural and Forestry Technology Co., Ltd., Xi’an 710005, China; lxy97@163.com (X.L.); shenli1963@163.com (L.S.)

**Keywords:** *Idesia polycarpa*, *Agrobacterium rhizogenes*, hairy roots, oil-bearing woody plant

## Abstract

*Idesia polycarpa* is a promising woody oilseed species because of its high oil yield. However, its use is greatly limited due to the lack of varieties with good qualities; additionally, gene function has been less studied in this plant because an efficient transformation method has not been established yet. In this study, we established a rapid and efficient hairy root transformation method by infecting the whole seedling, the rootless seedling, and the leaf petiole with *Agrobacterium rhizogenes* using different infection methods. Among these transformation methods, a higher transformation efficiency was obtained using the whole seedling, which could reach up to 71.91%. Furthermore, we found that the seedling age significantly affected the transformation efficiency, either using whole or rootless seedlings. Additionally, we found that the transgenic roots could regenerate transgenic shoots. Taken together, our study lays the foundation for future study and for genetically modifying wood traits in the future.

## 1. Introduction

*Idesia polycarpa* Maxim., a dioecious plant, is a woody oilseed tree primarily found in Asian countries, including China, Japan, and Korea [1]. Its fruits can be processed to yield edible vegetable oil [2], which is rich in unsaturated fatty acids (FAs), including palmitoleic acid (C16:1), oleic acid (C18:1), and linoleic acid (C18:2) [1,3]. This woody plant attracts significant attention due to its high oil content and nutritional value [4], with potential applications in healthcare products [4,5], industrial oils [1], cosmetic preparations [6], landscape gardening [2], etc. However, due to the lack of an efficient regeneration and transformation method for *I. polycarpa*, its industrial use and gene function study are greatly limited.

Various transformation methods have been developed for the delivery of exogenous genes into plant cells. The most widely used transformation method is mediated by *Agrobacterium tumefaciens*. However, this transformation method is time-consuming and inefficient, since the transgenic plants are usually regenerated from transformed cells via the tissue culture stage. Moreover, successful *Agrobacterium* transformation is largely dependent on species, genotype, and explant type [7]. Particle bombardment and electroporation were also used for gene delivery. However, these methods cannot generate stably transformed plants; thus, gene function cannot be genetically analyzed. Besides the above-mentioned transformation methods, *Agrobacterium rhizogenes*-mediated hairy root transformation is being widely used for some plants, including soybean [8,9], cotton [10], pigeon pea [11,12], cannabis [13], *Sphaeralcea angustifolia* [14], tea plant [15], citrus [16], Tung Tree [17], etc. This method involves the integration of Ri plasmid fragments into the host plant genome by infecting plant leaves or stems. With this approach, the production of transgenic hairy roots can either be achieved via the traditional tissue culture process or, more conveniently, circumvent the need for tissue culture altogether, thus quickly regenerating the transgenic plants [18,19,20]. Taken together, this method is simple, time-saving, and also avoids damaging plant tissues. 

Due to the high industry values of *I. polycarpa*, here, we initially evaluated the effects of different explant types on hairy root induction, including whole seedlings, rootless seedlings, and petioles. We also checked the impacts of development stages on the Agrobacterium-mediated transformation of *I. polycarpa*, such as non-, semi- and fully lignified stages. Additionally, we successfully induced transgenic buds from these genetically modified roots, ultimately yielding transgenic *I. polycarpa* plants. Our study is of great value for genetically modifying wood traits and for conducting scientific research on woody plants in the future. 

## 2. Results

### 2.1. Establishment of Transformation System Using Whole Seedlings as Explants

**Figure 1:**
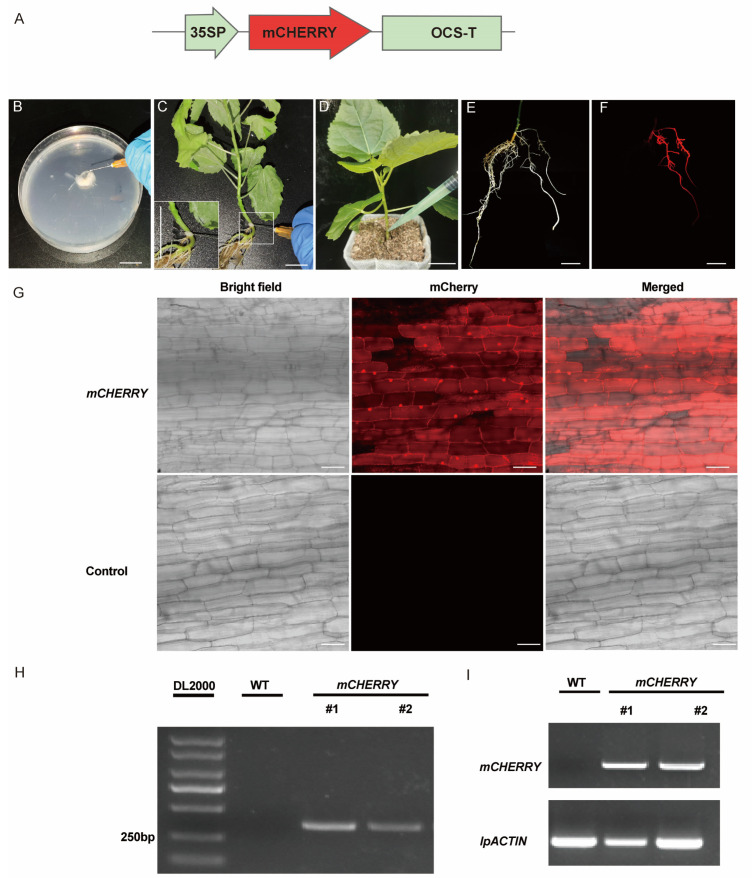
*Agrobacterium rhizogenes*-mediated transformation procedure using whole seedlings and transgenic root identification. (**A**) Schematic diagram of *PFGC-mCHERRY*. (**B**–**F**) Workflow of generation of transgenic roots. In short, bacteria clumps were prepared, and bacterial clumps were collected using the needle (**B**). Bacteria clumps were applied to the junction sites between the hypocotyl and roots (**C**). Bacterial solution was injected into the soil around the puncture sites (**D**). Hairy roots emerged around the puncture sites (**E**), which were further detected using a portable fluorescent lamp (**F**). Bars represent 1 cm. (**G**) Signals observed in transgenic roots expressing mCHERRY using a confocal laser scanning microscope with an excitation wavelength of 633 nm. Bars represent 50 µm. (**H**) PCR analysis of the insertion of *mCHERRY* into the genome. (**I**) RT-PCR analysis of *mCHERRY* expression in non-transformant and transgenic roots.

In this study, we first used the whole seedling for the transformation, and the strain used for the transformation was *Agrobacterium rhizogenes* K599 carrying *pFGC-mCHERRY* construct (Figure 1A). The base stem was punched using a syringe, and the bacteria solution was then applied to the wounding sites (Figure 1B–F). To maintain high humidity levels, these infected plants were always covered with a transparent plastic lid. After two to three weeks, a small area of callus emerged around the injection sites of the seedlings. After another four to five weeks, hairy roots appeared and exhibited a robust growth state later. 

Previous studies have shown that hairy roots originate from cortical cells, and the proportion of these cells decreases with the physiological age of the seedlings [21]; it is possible that the maturity state of the seedlings might affect the sensitivity of these cells to *Agrobacterium rhizogenes*, thereby influencing the transformation efficiency. To optimize the transformation efficiency, 20-, 30-, 40-, and 100-day-old seedlings were used for transformation. The induction rates of hairy roots were different among the tested plants with developmental stages (Table 1). Root induction rates were higher in the younger explants, including 20-, 30-, and 40-day-old seedlings, while lower in old explants, such as 100-day-old explants. Moreover, the transformation efficiency varied among plants at different development stages. The transformation rate of 40-day-old seedlings was highest (up to 71.91%), while the transformation rate of 100-day-old seedlings was only zero (Table 1), and that of 20 and 30-day-old seedlings were 49.94% and 58.09%, respectively. Plants containing transgenic roots account for 59.87% of total transformed plants (Table S1). The ratio of transgenic roots per seedling ranged between 36.47% and 53.07% (Table S2). 

To identify the transgenic roots, we usually checked the fluorescent signals using a portable fluorescence detection instrument equipped with a red filter, since the construct carrying *mCHERRY* was used for this study (Figure 1A), and those successfully transformed hairy roots exhibited red signals (Figure 1F). To confirm the reliability of the detection method, we also identified the transgenic roots using other methods, including confocal microscopy, PCR, and RT-PCR methods. Under confocal microscopy, strong signals were detected in the transformed roots’ epidermis cells expressing *mCHERRY* but were not seen in the non-transformed ones (Figure 1G). We also detected the *mCHERRY* gene using PCR analysis. Strong bands were detected in the transgenic roots but were not present in the non-transformed roots (Figure 1H). Meanwhile, we detected the transgene expression levels using RT-PCR analysis (Figure 1I). Our results showed that the bands from the *mCHERRY* gene were only detected in the transgenic roots, indicating that this gene was expressed in the transgenic roots. Taken together, these results confirmed the reliability of the detection method mediated by the portable fluorescent lamp.

### 2.2. Establishment of Transformation System Using Rootless Seedlings as Explants

**Figure 2:**
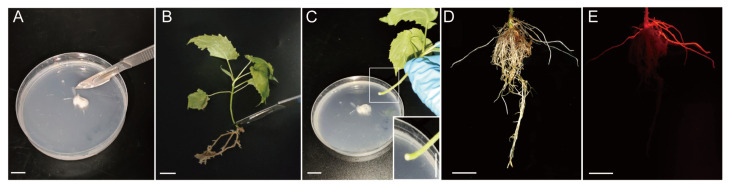
*Agrobacterium rhizogenes*-mediated transformation procedure using rootless seedlings. In short, bacteria colonies were piled up using a surgical blade (**A**); meanwhile, the seedling roots were removed using a blade (**B**). The cutting site of the rootless seedling was dipped into the bacterial clumps (**C**). The induced hairy roots appeared around the cutting sites (**D**), and the transgenic roots were detected using a portable fluorescent lamp (**E**). Bars represent 1 cm.

To find out the most suitable explants for transformation, we also chose the rootless seedlings as explants. The primary roots were removed at the stem base near the stem–root junctions, and the cutting sites of the plantlets were dipped into K599 colony clumps (Figure 2). Hairy roots were induced at 30 days after being dipped, and the transgenic roots were checked using the portable fluorescent lamp. Seedlings of different developmental ages were used in this study, and the average hairy root induction rate was 71.35%, and the plants containing transgenic roots accounted for about 32.58% of the total infected plants (Table S1). The ratios of transgenic roots per seedling are between 26.5% and 34.76% (Table S2). The transformation efficiency of 20-, 30-, 40- and 100-day-old seedlings is 9.72%, 30.00%, 58.22%, and 0.05%, respectively (Table 2). Taken together, these findings showed that the seedling age affects the transformation efficiency either using whole seedlings or rootless seedlings.

### 2.3. Establishment of Transformation System Using Leaf Petioles as Explants

**Figure 3:**
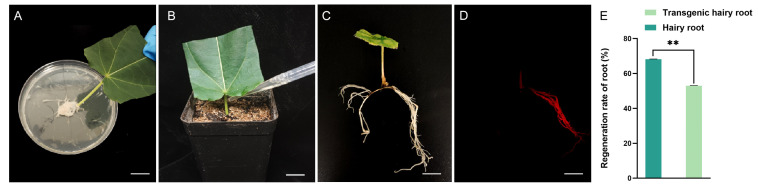
Transformation procedure using leaves as explants and leaf transformation efficiency. (**A**–**D**) Transformation procedure of leaf explants. Bars represent 1 cm. Briefly, petioles of leaves with the distal parts removed were dipped into bacterial clumps (**A**) and then inserted into wet vermiculites (**B**). The induced hair roots emerged around the petioles about 6 weeks after infection (**C**), and the fluorescent signals of transgenic roots were detected by the portable fluorescent lamp (**D**). (**E**) Leaf transformation efficiency. The transformation efficiency was calculated based on 21 leaves, and experimental data were repeated three times. The data were statistically analyzed using SPSS software version 27 for *t*-tests, ** indicate statistical significance (*p* < 0.05).

Due to the fact that many leaf explants can be obtained from a single plant, as compared to the rooted or rootless seedlings, we also utilized them as explants for *Agrobacterium rhizogenes* transformations. To increase leaf viability and reduce water evaporation, we first removed the distal parts of the leaves and reserved the proximal parts with the leaf petiole. The petioles were dipped into the *Agrobacterium rhizogenes* K599 clumps and then incubated in the trays filled with vermiculite (Figure 3A,B). The trays were always covered with cling film for moisture retention. After 2–3 weeks, small amounts of white calli appeared around the infection sites. After 6 weeks, hairy roots were seen around the infection sites of leaf petioles; the hairy root induction rate was 68.25%, and the leaf transformation efficiency was up to 53.48% (Figure 3C–E).

### 2.4. Regeneration of Transgenic Shoots from Transgenic Roots

**Figure 4:**
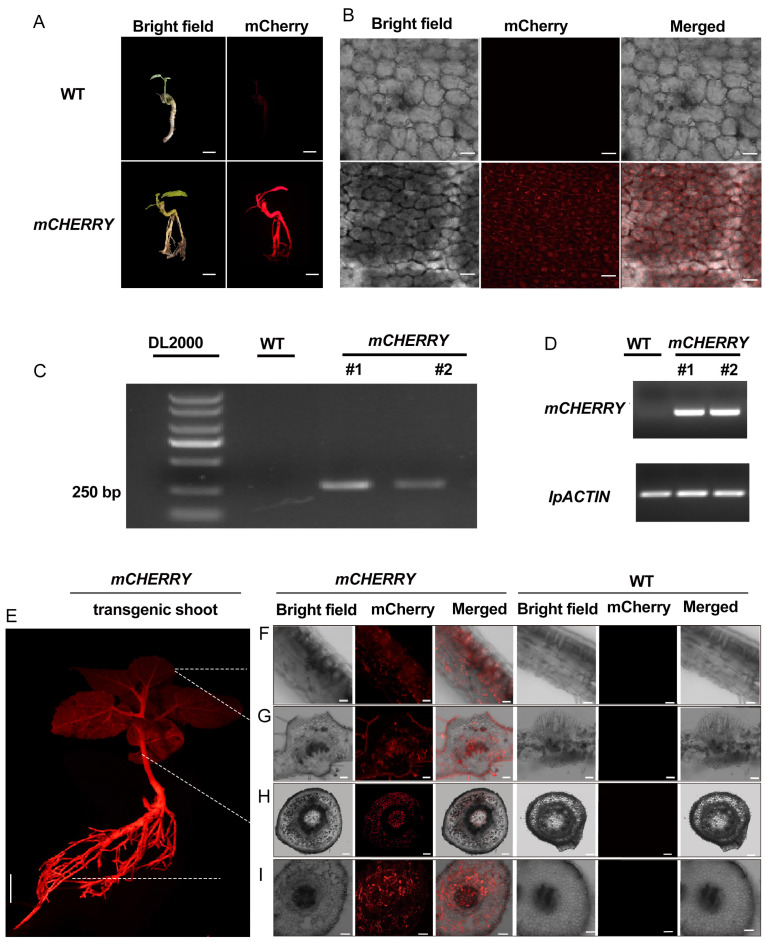
Transgenic shoot regeneration from transgenic roots and the identification of transgenic seedlings. (**A**) Shoots regenerating from transgenic roots. The plants were observed in a bright field (**left**) and using a portable fluorescent lamp (**right**). Upper panel, non-transformed plant; lower panel, plant transformed with *35Spro*: *mCHERRY*. Bars represent 1 cm. (**B**) Signals detected in transgenic shoots and untransformed shoots using a confocal laser scanning microscope with the excitation wavelength at 633 nm. Bars indicate 20 µm. (**C**) RT-PCR analysis of *mCHERRY* expression in non-transformant and transgenic shoots. Total RNA was extracted from the transgenic leaves. *IpACTIN* was used as the internal control. (**D**) PCR analysis of *mCHERRY* inserted into the genome. Genomic DNA was extracted from leaves of untransformed and transformed shoots. (**E**) Six-month-old whole transgenic plants under a portable fluorescent lamp. Bars indicate 1 cm. (**F**–**I**) Cross-sections of leaf blades (**F**), leaf midveins (**G**), stems (**H**), and roots (**I**) of transgenic plants and untransformed plants (WT). Bars in (**F**–**I**) represent 20, 50, 200, and 100µm.

In our study, we noticed that *I. polycarpa* roots are prone to generating new shoots without the addition of hormones. Thus, we cut the transgenic roots carrying the *mCHERRY* gene into segments and placed them in vermiculite under strong light and high humidity. After around 30 days, shoots sprouted out of the transgenic roots. To confirm if the newly emerged branches were transgenic, we used the portable fluorescent lamp to detect the fluorescent signals in these newly emerged leaves. The signals were detected in the leaves growing from the transgenic roots but were not seen in the untransformed leaves (Figure 4A,B). To confirm the reliability of the detection method, we also examined the transgenic shoots using other methods, including confocal microscopy, PCR, and RT-PCR methods. The bands representing the *mCHERRY* gene were only detected in the transgenic buds, whereas they were undetectable in the non-transformed buds (Figure 4C,D). These results indicate that the *mCHERRY* gene was expressed in the transgenic buds. Here, a total of 54 root segments were used for this study, among which 8 segments successfully generated new shoots. This result indicates that the transgenic shoots were generated from transgenic roots that bypass tissue culture. To examine where the transgenes were expressed, we also checked the fluorescence in 6-month-old plants. We found that the fluorescence was detected in all parts of some transgenic plants, including the leaves, stems, and roots (Figure 4E–I). To further check which tissues the signals had accumulated in, we obtained cross-sections of leaves, stems, and roots and found that the signals were almost detected in most tissues (Figure 4F–I). Taken together, the method we established enables the possibility of modifying *I. polycarpa* fruit traits using gene editing technology. 

## 3. Discussion

In this study, we successfully obtained the transgenic roots of *I. polycarpa* using *Agrobacterium rhizogenes*-mediated genetic transformation technology. This method has been successfully applied to some woody plants as well, since it overcomes many challenges that arise from the *Agrobacterium tumefaciens*-mediated transformation method, including long transformation periods, rigid sterile conditions, the limitations of species and genotype, etc. [21]. Moreover, this technology can be flexibly applied to different types of explants, including leaves, stem segments, seeds, and rootless seedlings [22,23,24,25]. In our study, using three types of explants, including whole seedlings, rootless seedlings, and leaves, we successfully obtained transgenic roots. Moreover, the transgenic roots could generate transgenic shoots. Thus, the establishment of the transformation procedure in *I. polycarpa* laid a solid foundation for future gene function identification and industrial use. 

Root regeneration efficiency is crucial for *Agrobacterium rhizogenes*-mediated hairy root transformation. Our research has revealed that this efficiency varies significantly among explants at different developmental stages (Table 1 and Table 2). Either using whole seedlings or rootless seedlings as explants, the root induction rates of young explants including 20-, 30-, and 40-day-old seedlings were significantly higher than that of 100-day-old seedlings (Table 1 and Table 2), indicating that the root regeneration efficiency is closely related to the developmental stages. This result is consistent with previous studies [26,27,28] that obtained similar results using Arabidopsis leaf petioles as explants [26]. Moreover, these studies identified that the decreasing root regeneration rates of the older explants are due to the reduction in the ability to biosynthesize auxin during maturation [26]. The reduced auxin production ability in older explants is attributed to two possible reasons. The first reason is due to the ethylene production induced by wounding. In our study, and previous studies, the explants were wounded either by punching or cutting before transformation (Figure 1 and Figure 2). Wounding treatments do not only induce auxin accumulation, but also trigger ethylene production and activate the expression levels of transcription factor *ETHYLENE INSENSITIVE 3* (*EIN3*) [27]. Ethylene treatment, or the activation of EIN3, is identified to suppress root regeneration ability. Relative to young explants, the older ones contain higher EIN3 levels, thus inhibiting root regeneration efficiency by suppressing the expression of two key genes, *WUSCHEL RELATED HOMEOBOX 11* (*WOX11*)/*WOX12* and *WOX5*/*WOX7*, which are required for de novo root regeneration [27]. The other possible reason is related to the regulatory roles of the miR156-SPLs-AP2/ERFs pathway. Transcription factors AP2/ERFs are identified to promote root regeneration by inducing auxin biosynthesis but are negatively regulated by *SPLs*. *SPLs* are preferentially accumulated in older explants, thereby suppressing root regeneration by repressing the activity of AP2/ERFs [28]. Besides the above-mentioned two possible reasons, we speculated that the root regeneration ability of explants with different developmental ages might be closely associated with the proportion of cambium cells. Those young explants exhibiting higher root regeneration ability usually contain a higher proportion of cambia cells, whereas the old ones contain fewer cambia cells. Therefore, the old explants display decreased root regeneration efficiency. 

In our study, we noticed that a higher transformation efficiency was obtained by 30- 40-day-old explants, while the younger (20-day-old seedlings) or older explants (100-day-old seedings) showed a lower transformation efficiency (Table 1 and Table 2). One possible reason for this is that 20-day-old explants are too vulnerable to withstand the *Agrobacterium* infection, though they have similar root induction rates to 30- or 40-day-old explants. However, the low transformation efficiency of 100-day-old explants might be due to their low root regeneration ability (Table 1 and Table 2). 

Our study showed that the transgenic hair roots of *I. polycarpa* could produce the shoots, though this conversion of roots to shoots requires a relatively long period of time (Figure 4). The method we used is similar to the recently reported cut-dip-budding (CDB) method [20]. Using the CDB method, transformants are successfully obtained from some other plants, including two herbaceous plants (*Taraxacum koksaghyz* and *Coronilla varia*), a tuberous root plant (sweet potato), and three woody plant species (*Ailanthus altissima*, *Aralia elata*, and *Clerodendrum chinense*) [20]. Until now, the acquisition of transgenic plants of *I. polycarpa* or other plants has been mainly based on the root-suckering capability of plants. In addition, we found that light plays a crucial role in the conversion of roots to shoots of *I. polycarpa*, since the buds of *I. polycarpa* preferentially often emerge on the root parts exposed to light. That is consistent with previous studies’ observations that light plays a key role in shoot apical meristem growth and leaf initiation by regulating both cytokinin signaling and auxin transport [29,30]. Anyway, the enhancement of bud regeneration ability in non-sterile environments will facilitate the establishment of genetic transformation systems in woody plants.

## 4. Conclusions

Here, we first established the *Agrobacterium rhizogenes*-mediated transformation methods of *I. polycarpa* using whole seedlings, rootless seedlings, or petioles as explants, respectively. Among the different explants, the whole seedlings display a higher transformation efficiency. We also found that the developmental age has effects on transformation efficiency. Finally, we successfully obtained transgenic shoots from transgenic roots. Our study will be helpful for genetically modifying woody plants in the future.

## 5. Materials and Methods

### 5.1. Plant Materials and Growth Conditions

The seeds of *I. polycarpa*, obtained from Hanzhong, Shaanxi Province, were sown in nutrient-rich soil in big trays (55 cm × 28 cm). The seedlings were germinated in Petri dishes for 10 days on wet filter paper and grown in a growth chamber with two fully expanded true leaves, and were transferred to small individual pots (7 cm × 7 cm). One *I. polycarpa* seedling was planted in each pot. The plants grew in a greenhouse with temperatures set between 22 and 26 °C with a 16 h light/8 h dark photoperiod.

### 5.2. Preparation of Agrobacterium rhizogenes

The plasmid carrying *pFGC-mCHERRY* was transformed into the *Agrobacterium rhizogenes* strain K599 using the freeze–thaw method. A freshly single colony growing on a solidified LB medium containing appropriate antibiotics was inoculated into a 3 to 5 mL LB liquid medium containing kanamycin, rifampicin, and 10 mM calcium chloride. The cultures were shaken at 28 °C for 12 h. Then, 300 µL of bacterial cultures was plated onto LB agar plates containing appropriate antibiotics and cultured for another day. The colonies were piled up for injection. To prepare the *Agrobacterium* solution for irrigation, the overnight cultures were diluted in LB media at a 1:1000 ratio and then subcultured for another day until OD600 ranged between 0.8 and 1.0. 

### 5.3. Agrobacterium rhizogenes Infection

For the *I. polycarpa Agrobacterium rhizogenes* infection, some simple modifications were made to the cut-dip-budding (CBD) method [20]. Specifically, the stems near the roots (about 1–2 cm above the roots) were punched with a syringe, or the primary roots of the seedlings were cut off, or freshly picked leaves containing petioles were used. *Agrobacterium* K599 colonies screening on solidified LB plates containing appropriate antibiotics were applied to the wound sites. The infected seedlings were planted in a pot filled with wet sterile vermiculite and irrigated with 3–5 mL of *Agrobacterium* solution. Finally, the pots were covered with a transparent lid to maintain high humidity levels, and hairy roots grew up approximately 5–6 weeks later. PCR analysis, gene expression analysis, and fluorescence observation were conducted to determine the generation of transgenic roots.

### 5.4. Regeneration of Transgenic Shoots

The lignified transgenic roots were cut into multiple segments with a length of 5–6 cm. After that, the segments were vertically inserted into the sterilized vermiculite and incubated in a greenhouse until the shoots emerged. The treated explants were cultured at 22–26 °C with a 16 h light/8 h dark cycle until buds developed from the root cuttings (approximately 1 month). Putative transgenic buds were then transferred to the potted soil (nutrient soil: vermiculite = 1:1) and grown under normal growth conditions.

### 5.5. Fluorescence Observation

The transformed hairy roots were thoroughly cleansed, and the mCHERRY fluorescent signals were inspected using a portable excitation light source (LUYOR-3410GR) paired with specific glasses featuring LUV-50A filters. For further analysis, the mCHERRY signal was detected in intact leaves or freehand sections of leaf blades and midveins, the stems, and the roots using a confocal fluorescence microscope (LSM980, Zeiss, Oberkochen Germany) equipped with a 610 nm emission filter, and a 587 nm excitation filter was utilized. 

### 5.6. PCR Analysis

DNA was extracted from transgenic roots or shoots using the CTAB method. PCR amplification was performed using 2×Taq Master Mix reagents (Vazyme, Nanjing, China). To detect the inserts of *mCHERRY* gene fragments in transgenic roots and transgenic shoots, a PCR was performed using specific primers *mCHERRY*-F: 5′-ATGGGAGCCAATGGAGTG-3′ and *mCHERRY*-R: 5′-TCAAAACTGGTTCCGGTAC-3′, respectively [17].

### 5.7. RT-PCR Analysis

Total RNA was extracted from transgenic roots or shoots using the Trizol reagent (Simgen, Hangzhou, China). Reverse transcription was carried out using HiScriptII1st Strand cDNA Synthesis Kit (Vazyme, Nanjing). RT-PCR analysis was performed using the above-mentioned primers, *mCHERRY*-F and *mCHERRY*-R. *IpACTIN* was used as the internal control, of which the primers are *IpACTIN*-qPCR-F: 5′-AAGACCTACACCAAGCCGAA-3′ and *IpACTIN*-qPCR-F: 5′-CTCCGCACTCAGCATTAGGACA-3′, respectively.

### 5.8. Statistical Analysis

All experiments were repeated three times. Each statistical analysis was carried out using SPASS software 27 for *t*-tests. We calculated the induction rate (%) of hairy roots by using the ratio of the number of plants producing hairy roots to the total number of survived injected plants and separately calculated the induction rate (%) of transgenic hairy roots based on the ratio of the number of transgenic hairy root plants to the total number of hairy root plants.

## Figures and Tables

**Table 1:** Hairy root induction rates and transformation efficiency of whole seedlings at different ages.

Seedling Age	Rooting Rates (%)	Transgenic Rates (%)
20 days old	96.45 ± 1.86 a	49.94 ± 4.52 b
30 days old	100 ± 0 a	58.09 ± 4.10 ab
40 days old	98.25 ± 1.67 a	71.91 ± 5.97 a
100 days old	10.88 ± 1.00 b	0.00 ± 0 c

Note: The data presented are averages ± SD, and statistical analysis was conducted using SPSS software 27 for *t*-tests, different lowercase letters indicate that the difference is significant (*p* < 0.05).

**Table 2:** Adventitious root induction rates and transformation efficiency of rootless seedlings with different ages.

Seedling Age	Rooting Rates (%)	Transgenic Rates (%)
20 days old	97.92 ± 2.00 a	9.72 ± 5.00 c
30 days old	98.33 ± 1.67 a	30.00 ± 10.00 b
40 days old	100 ± 0 a	58.22 ± 2.50 a
100 days old	13.6 ± 4.48 b	0.05 ± 0.03 c

Note: The data presented are averages ± SD, and statistical analysis was conducted using SPSS software 27 for *t*-tests, different lowercase letters indicate that the difference is significant (*p* < 0.05).

## Data Availability

Data are contained within the article and Supplementary Materials.

## Supplementary Materials

The following supporting information can be downloaded at: https://www.mdpi.com/article/10.3390/plants13131791/s1, Table. S1 Average hairy root induction rates and transformation efficiency from whole seedlings and rootless seedings. Table. S2 Induction of transgenic roots contained in each seedling from whole seedlings and rootless seedings.
