# Supplementary material for: A Highly Efficient Agrobacterium rhizogenes-Mediated Hairy Root Transformation Method of Idesia polycarpa and the Generation of Transgenic Plants
Source: Plants (Basel). 2024 Jun 28;13(13):1791. doi: 10.3390/plants13131791 (PMC11244318; doi:10.3390/plants13131791)
Supplement: Supplementary file 1 [file plants-13-01791-s001.zip › plants-3021241-supplementary.pdf]

Table.S1 Average hairy root induction rates and transformation efficiency from whole seedlings and rootless seedings

Infection ways	Numbers of infected plants	Numbers of surviving infected plants	Numbers of hairy roots plants	Number of positive plants	Average hairy roots efficiency (%)	Average transformation efficiency (%)
Whole seedlings	231	188	152	91	80.85%	59.87%
Rootless seedlings	252	185	132	43	71.35%	32.58%
leaf petioles	63	63	43	23	68.25	53.48

Table.S2 Induction of transgenic roots contained in each seedling from whole seedlings and rootless seedings

Infection ways	No. of groups	Numbers of hairy roots roots	Number of positive roots	transformation efficiency	Average transformation efficiency (%)
Whole seedlings	1	10	2	20.00%	39.05%
		7	1	14.29%	
		6	3	50.00%	
		4	2	50.00%	
		3	2	66.67%	
		3	1	33.33%	
	2	5	1	20.00%	53.07%
		3	1	33.33%	
		9	5	55.56%	
		7	3	42.86%	
		6	4	66.67%	
		6	6	100.00%	
	3	6	2	33.33%	36.47%
		4	1	25.00%	
		7	4	57.14%	
		12	5	41.67%	
		12	5	41.67%	
		5	1	20.00%	
Rootless seedlings	1	7	2	28.57%	34.76%
		10	3	30.00%	
		8	4	50.00%	
		6	2	33.33%	

	6	1	16.67%	
	6	3	50.00%	
2	6	1	16.67%	26.50%
	6	2	33.33%	
	10	2	20.00%	
	8	3	37.50%	
	11	2	18.18%	
	12	4	33.33%	
3	5	1	20.00%	26.67%
	5	1	20.00%	
	5	2	40.00%	
	10	3	30.00%	
	6	1	16.67%	
	6	2	33.33%	
